# Cell type-specific polygenic burden modulates exercise effects in schizophrenia patients: further evidence on volumes of hippocampal subfields

**DOI:** 10.1007/s00406-024-01841-9

**Published:** 2024-07-05

**Authors:** Sergi Papiol, Lukas Roell, Isabel Maurus, Dusan Hirjak, Daniel Keeser, Andrea Schmitt, Andreas Meyer-Lindenberg, Peter Falkai

**Affiliations:** 1 Max Planck Institute of Psychiatry, Kraepelinstrasse 2-10, 80804 Munich, Germany; 2 Institute of Psychiatric Phenomics and Genomics (IPPG), LMU University Hospital, LMU Munich, Munich, Germany; 3 Department of Psychiatry and Psychotherapy, LMU University Hospital, LMU Munich, Munich, Germany; 4 Medical Faculty Mannheim, Central Institute of Mental Health, Heidelberg University, Heidelberg, Germany; 5 German Centre for Mental Health (DZPG), Partner site Mannheim/Heidelberg/Ulm, Germany; 6 NeuroImaging Core Unit Munich (NICUM), University Hospital LMU, Munich, Germany; 7 Munich Center for Neurosciences (MCN), LMU Munich, Munich, Germany; 8 Laboratory of Neuroscience (LIM27), Institute of Psychiatry, University of Sao Paulo, São Paulo, Brazil; 9 German Centre for Mental Health (DZPG), Partner site Munich/Augsburg, Germany

**Keywords:** Physical activity, Polygenic risk score, Schizophrenia, Hippocampus, Oligodendrocyte precursor cell

Despite extensive efforts to tackle schizophrenia symptoms, evidence shows that current treatment options just achieve partial success to improve the clinical outcome in many of these patients [1]. This limited efficacy is especially pronounced if negative symptoms [1] or cognitive deficits [2] are considered regarding the functional recovery of these patients. Exercise holds promise as an adjunt therapy for improving those clinical symptoms poorly managed by current pharmacological interventions [3]. In this vein, a series of multicenter studies have provided compelling evidence of the beneficial effects of physical activity on cognition, psychopathology, and social functioning in multi-episode schizophrenia patients [4–6].

However, little is known about the biological mechanisms linking physical activity and clinical outcome. Metanalyses in the general population have shed light on this question, suggesting that aerobic exercise could significantly increase hippocampal volume [7]. These findings align with the several studies on schizophrenia patients undergoing aerobic exercise that observed increases in hippocampal / hippocampal subfield volumes, although some conflicting evidence has also been reported [4, 8, 9] (see also preprint 10.31219/osf.io/y2phs).

Another common observation in the field is that not all patients have a positive response to the same pattern of physical activity. While the explanation for this interindividual variability is likely complex and multifactorial, recent advances in genomics have provided initial clues about the role of biological factors in these individual responses. Particularly, a previous study reported the role of schizophrenia polygenic risk (the so-called polygenic risk scores-PRS) associated with oligodendrocyte precursor cells (OPC) and radial glia (RG) on the volumetric hippocampus changes in CA4/Dentate Gyrus (DG) in patients undergoing aerobic exercise [10]. In this study, a larger genetic burden was associated with a lack of effect of aerobic exercise on the left CA4/DG hippocampus subfields or even a decrease of the volume in this brain area.

In the present study, we have analyzed the previously reported effects of the polygenic scores on a new cohort of schizophrenia patients of a randomized clinical trial performing aerobic endurance training (AET) or flexibility, strengthening, and balance training (FSBT) during six months [6]. A subset of this cohort with genetic and imaging data available was used for our genetic study (AET *N* = 6; FSBT *N* = 17). Details on imaging protocols are available in the original publication [6]. We estimated the polygenic burden of schizophrenia specific for oligodendrocyte precursor cells (PRS-OPC), radial glia (PRS-RG), mature oligodendrocytes (PRS-OLI), interneurons (PRS-INT), pyramidal CA1 neurons (PRS-PYR), and dopaminergic neurons (PRS-DOP) in these participants. Written consent was obtained for all the samples, and the study was approved by the ethical committees of the participating centres.

Polygenic Scores (PGS) were calculated using the imputation dosage for each risk allele based on the results of latest schizophrenia GWAS (https://figshare.com/articles/dataset/scz2022/19426775). Integration of cell type specific gene-sets in the PRS calculation was performed as described elsewhere [10]. PRS-CS tool (https://github.com/getian107/PRScs) was used to infer posterior SNP effect sizes under continuous shrinkage priors and estimate the global shrinkage parameter (φ) using a fully Bayesian approach.

Linear regression models did not detect any influence of PRS-OPC or PRS-RG on the volumetric changes in CA4 or DG after 6 months of AET and FBST grouped together (all P-values > 0.34). All models were corrected for age, sex, AET/FBST group, chlorpromazine equivalents, and the first two ancestry components. However, in AET patients Spearman’s rank correlation analyses observed significant or borderline significant correlations between PRS-OPC and the volume change in left CA4 (S = 66, P-value = 0.033, rho = -0.89, 95% CI [-0.99, -0.26]) and left DG (S = 64, P-value = 0.058, rho = -0.83, 95% CI [-0.98, -0.05]) (Fig. 1A and 1B). AET participants with a larger genetic burden tend to have a reduction of hippocampus subfields volume after exercise intervention, in a similar fashion as in the aforementioned study [10]. Despite showing a similar trend, results of PRS-RG on left CA4 and left DG (Fig. 1C and 1D) did not show a significant Spearman’s rank correlation (all P-values > 0.14). No effect was observed for the genetic burden associated with other cell types (PRS-OLI, PRS-INT, PRS-PYR, or PRS-DOP).

**Fig. 1:**
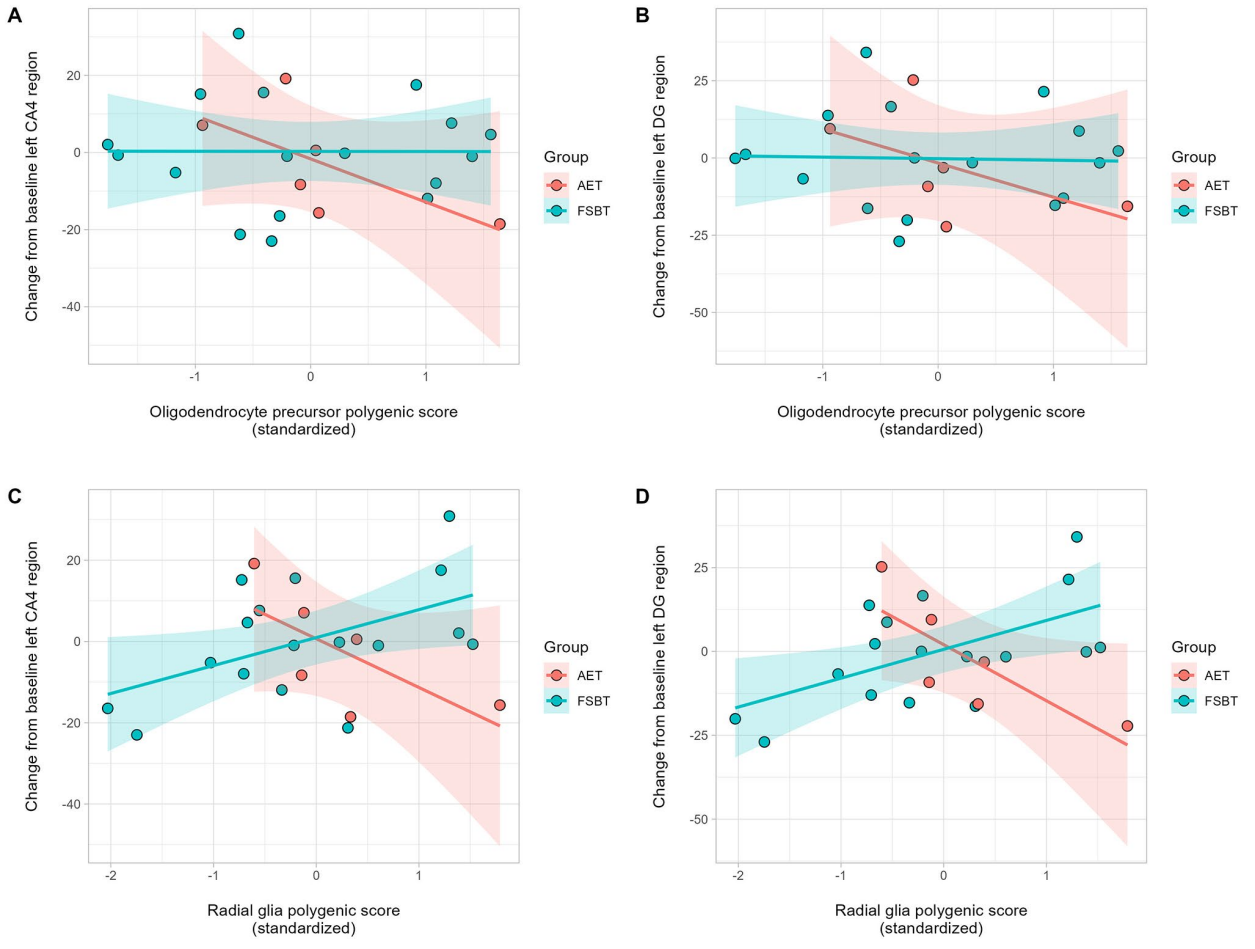
**(A-B)** Scatterplot showing the relationship between the PRS-OPC and the change from baseline (V1) in the volume of the left hippocampal subfields CA4 and dentate gyrus (DG) after 6 months of aerobic exercise. **(C-D)** Scatterplot showing the relationship between the PRS-RG and the change from baseline (V1) in the volume of the left hippocampal subfields CA4 and dentate gyrus (DG) after 6 months of aerobic exercise. In all panels, positive values in the y-axis indicate a gain in volume after 6 months; and positive values in the x-axis, a higher genetic risk burden. Also shown are regression line and 95% confidence intervals based on the predicted means from the regression line for each group, Aerobic Endurance Training (AET) or flexibility, strengthening, and balance training (FSBT)

Taken together, our results reinforce the notion of a modulatory role of the polygenic burden for schizophrenia associated to OPC and, to a lesser extent, RG, on the effects of AET on hippocampal subfields volumes. Our results also suggest that the type and intensity of physical activity (AET vs. FBST) are important regarding their impact on brain volumetric changes. However, the small sample size warrants further replication in larger cohorts in order to confirm or discard the relevance of this genetic effect.

## References

[1] Fusar-Poli P, Papanastasiou E, Stahl D et al (2015) Treatments of negative symptoms in Schizophrenia: Meta-Analysis of 168 randomized placebo-controlled trials. Schizophr Bull 41:892–899. 10.1093/schbul/sbu170 25528757 10.1093/schbul/sbu170 PMC4466178

[2] Nielsen RE, Levander S, Kjaersdam Telléus G et al (2015) Second-generation antipsychotic effect on cognition in patients with schizophrenia–a meta-analysis of randomized clinical trials. Acta Psychiatr Scand 131:185–196. 10.1111/acps.12374 25597383 10.1111/acps.12374

[3] Dauwan M, Begemann MJH, Slot MIE et al (2021) Physical exercise improves quality of life, depressive symptoms, and cognition across chronic brain disorders: a transdiagnostic systematic review and meta-analysis of randomized controlled trials. J Neurol 268:1222–1246. 10.1007/s00415-019-09493-9 31414194 10.1007/s00415-019-09493-9 PMC7990819

[4] Pajonk F-G, Wobrock T, Gruber O et al (2010) Hippocampal plasticity in response to exercise in schizophrenia. Arch Gen Psychiatry 67:133–143. 10.1001/archgenpsychiatry.2009.193 20124113 10.1001/archgenpsychiatry.2009.193

[5] Malchow B, Keller K, Hasan A et al (2015) Effects of endurance training combined with cognitive remediation on everyday functioning, symptoms, and Cognition in Multiepisode Schizophrenia patients. Schizophr Bull 41:847–858. 10.1093/schbul/sbv020 25782770 10.1093/schbul/sbv020 PMC4466186

[6] Maurus I, Roell L, Lembeck M et al (2023) Exercise as an add-on treatment in individuals with schizophrenia: results from a large multicenter randomized controlled trial. Psychiatry Res 328:115480. 10.1016/j.psychres.2023.115480 37716320 10.1016/j.psychres.2023.115480

[7] Li M-Y, Huang M-M, Li S-Z et al (2016) The effects of aerobic exercise on the structure and function of DMN-related brain regions: a systematic review. Int J Neurosci 1–16. 10.1080/00207454.2016.1212855 10.1080/00207454.2016.1212855 27412353

[8] Saviola F, Deste G, Barlati S et al (2023) The Effect of Physical Exercise on people with psychosis: a qualitative critical review of neuroimaging findings. Brain Sci 13:923. 10.3390/brainsci13060923 37371403 10.3390/brainsci13060923 PMC10295951

[9] Maurus I, Roell L, Keeser D et al (2022) Fitness is positively associated with hippocampal formation subfield volumes in schizophrenia: a multiparametric magnetic resonance imaging study. Transl Psychiatry 12:388. 10.1038/s41398-022-02155-x 36114184 10.1038/s41398-022-02155-x PMC9481539

[10] Papiol S, Keeser D, Hasan A et al (2019) Polygenic burden associated to oligodendrocyte precursor cells and radial glia influences the hippocampal volume changes induced by aerobic exercise in schizophrenia patients. Transl Psychiatry 9:284. 10.1038/s41398-019-0618-z 31712617 10.1038/s41398-019-0618-z PMC6848123

